# Structural, Surface and Optical Studies of m- and c-Face AlN Crystals Grown by Physical Vapor Transport Method

**DOI:** 10.3390/ma16051925

**Published:** 2023-02-25

**Authors:** Shuping Zhang, Hong Yang, Lianshan Wang, Hongjuan Cheng, Haixia Lu, Yanlian Yang, Lingyu Wan, Gu Xu, Zhe Chuan Feng, Benjamin Klein, Ian T. Ferguson, Wenhong Sun

**Affiliations:** 1 Research Center for Optoelectronics Materials and Devices, School of Physical Science and Technology, Guangxi University, Nanning 530004, China; 2 Center on Nano-Energy Research, Laboratory of Optoelectronic Materials & Detection Technology, School of Physical Science and Technology, Guangxi University, Nanning 530004, China; 3 Key Laboratory of Semiconductor Materials Science, Institute of Semiconductors, Chinese Academy of Sciences, Beijing 100083, China; 4 No. 46 Research Institute, China Electronics Technology Group Corporation, Tianjin 300220, China; 5 Department of Materials Science and Engineering, McMaster University, Hamilton, ON L8S4L7, Canada; 6 Southern Polytechnic College of Engineering and Engineering Technology, Kennesaw State University, Marietta, GA 30060, USA; 7 State Key Laboratory of Featured Metal Materials and Life-Cycle Safety for Composite Structures, Nanning 530004, China

**Keywords:** m- and c-face aluminum nitride, physical vapor transport method, spectroscopy, optical phonon modes, stress

## Abstract

Bulk aluminum nitride (AlN) crystals with different polarities were grown by physical vapor transport (PVT). The structural, surface, and optical properties of m-plane and c-plane AlN crystals were comparatively studied by using high-resolution X-ray diffraction (HR-XRD), X-ray photoelectron spectroscopy (XPS), and Raman spectroscopy. Temperature-dependent Raman measurements showed that the Raman shift and the full width at half maximum (FWHM) of the E_2_ (high) phonon mode of the m-plane AlN crystal were larger than those of the c-plane AlN crystal, which would be correlated with the residual stress and defects in the AlN samples, respectively. Moreover, the phonon lifetime of the Raman-active modes largely decayed and its line width gradually broadened with the increase in temperature. The phonon lifetime of the Raman TO-phonon mode was changed less than that of the LO-phonon mode with temperature in the two crystals. It should be noted that the influence of inhomogeneous impurity phonon scattering on the phonon lifetime and the contribution to the Raman shift came from thermal expansion at a higher temperature. In addition, the trend of stress with increasing 1000/temperature was similar for the two AlN samples. As the temperature increased from 80 K to ~870 K, there was a temperature at which the biaxial stress of the samples transformed from compressive to tensile stress, while their certain temperature was different.

## 1. Introduction

Aluminum nitride (AlN) is an important semiconductor material due to its excellent physical and chemical properties. Owing to its ultra-wide band gap, excellent ultraviolet transparency, and chemical stability, AlN can act as the substrate material for ultraviolet (UV)/deep ultraviolet (DUV) light-emitting diodes (LEDs), UV laser diodes (LDs) and solar-blind UV detectors applications and may meet the application requirements of microelectromechanical systems (MEMS) and high-temperature, high-power, high-frequency, and radiation-resistant devices [1,2,3,4,5,6,7]. The direct band gap of AlN is 6.2 eV at room temperature (RT), and it plays an important role in deep-ultraviolet devices, such as AlN-based UV-LED, which has great potential in UV curing, UV medical treatment, UV catalysis, UV sterilization, UV communication, high-density storage, etc. [8] In addition, the AlN ternary or quaternary and alloys (AlGaN, AlInN, or AlInGaN) can make the band gap of the material continuously adjust from 0.7–6.2 eV in the case of precisely controlling alloy compositions. Thus, the materials system can be applied in the full spectral region from UV, visible, and infrared wavelengths [9,10]. Furthermore, since AlN has a high carrier migration rate and ultra-high breakdown field strength, the overall performance of AlN-based high-power, high-frequency electronic devices have incomparable advantages and efficiency over other wide band gap semiconductor materials such as silicon carbide (SiC), etc. [11]. Therefore, AlN is still at the frontier of nitride semiconductor research [12,13,14].

The physical vapor transport (PVT) method of AlN crystal growth has great advantages in terms of growth rate and crystal quality over other epitaxy methods such as metal–organic chemical deposition (MOCVD) and halide vapor phase epitaxy (HVPE), and it is recognized as a prospect for preparing large-size, high-quality AlN bulk crystals [15,16,17]. However, the preparation of large-sized AlN single crystals still faces some challenges in terms of growth theory and process technology. In recent years, some institutions have carried out in-depth research on the growth of AlN single crystals and developed various growth processes. Shengtao Zhang et al. [18] investigated the effects of different temperature gradients for AlN growth and obtained an AlN crystal with a 52 mm diameter, a high-quality structure, an excellent uniformity, and lower thermal stress. C. Hartmann et al. [19] analyzed different growth conditions for the preparation of bulk AlN crystals, and they observed by simple diffusion and a step-flow model that the average growth rate increased with higher growth temperatures and higher thermal gradients. Danyang Fu et al. [20] studied Al-polar AlN single crystals up to 56 mm that were grown by the homoepitaxial PVT method. The full width at half maximum (FWHM) of 84–144 arcsec and 45–70 arcsec was obtained for the (0002) and (10−12) rocking curves, respectively. There were also some reports about the formation, density, and distribution of extended defects in a typical single-crystal AlN boule [21,22]. Obviously, the formation of type A LAGBs (low-angle grain boundaries) was relevant to the initial growth stage, and type B LAGBs consisted of threading edge dislocation (TED) arrays. In addition, the optical, piezoelectric, and thermal conductivity properties of AlN are key parameters to be considered in the implementation of devices. Wei Zheng et al. [23,24] studied the Raman tensor elements of the correlation Raman model from the AlN crystals by using angle-dependent polarized Raman spectroscopy and revealed that the angular dependencies of the A_1_ (TO), E_2_ (high), and E_1_ (TO) phonon modes show strongly anisotropic behavior. A. V. Inyushkin et al. [25] reported the thermal conductivity of bulk AlN single crystals in detail and found peculiarities in the low-temperature dependence of the thermal conductivity.

While some studies on AlN materials were conducted, mostly for epitaxial AlN [26,27,28,29,30,31,32,33], other properties of AlN crystal such as its spectroscopic characteristics with temperature are not well and thoroughly understood. The optical properties of semiconductors are useful for understanding the electronic band structure. The band structure and carrier behavior in materials are strongly influenced by lattice vibrations. Raman spectroscopy can reflect the lattice vibrations and can be used to measure the temperature of AlN-related materials and devices non-destructively and accurately. As a result, it is important to pay more attention to the effect of temperature on the Raman spectra. In this article, we systematically investigate the temperature-dependent Raman spectra of m-plane and c-plane AlN crystals grown by the PVT method, especially the temperature effects of the Raman shift, phonon FWHM, and optical phonon lifetime of the A_1_ (TO), E_2_ (high), E_1_ (TO), and A_1_ (LO) phonon modes. In addition, the transformation of the biaxial stress from compressive to tensile is revealed through spectroscopic methods. This research provides a basis for the non-invasive temperature measurement of III–V nitrides by Raman scattering during growth and processing.

## 2. Materials and Methods

Two AlN bulk crystals were grown, in a hot furnace with a dual-temperature zone of a metal system, by the PVT method [34,35,36]. Self-nucleation of the AlN crystal was realized by adjusting the temperature of the dual-temperature zone. The large AlN crystal was obtained by multi-time size-extension growth experiments. The substrate was polished tungsten, which was fixed at the top of the crucible. The raw material was AlN powder, which was sintered twice at high temperatures (2000 °C). The specific growth conditions were as follows: the growth atmosphere was N_2_ (purity: 99.999%), the growth pressure was 900–1200 mbar (1 mbar = 100 Pa), the temperature of the raw powder was 2250–2300 °C, and the temperature of the tungsten substrate was 2230–2280 °C. AlN single crystal with spontaneous nucleation was initially grown for 2–5 h, and, afterward, the growth of each AlN single crystal extension was carried out for 8–10 h.

The two typical AlN bulk samples denoted as m-bulk and c-bulk were chosen for study. The crystal orientation and structure of the AlN bulk samples were analyzed with PANalytical X’pert-Pro MRD high-resolution X-ray diffractometer (HR-XRD). The surface over-layers and the status of elements and compounds were probed by ESCALAB 250XI X-ray photoelectron spectroscopy (XPS), in which Al Kα (1486.7 eV) line was used as the excitation source. The XPS spectra were collected at a photoelectron take-off angle of 45° concerning the sample surface. The energy shifts of all XPS measurements were calibrated using C 1s electron peak at 284.8 eV as a reference. The narrow scan spectra were fitted to obtain the binding energies in different chemical states, using the fitting software XPSPEAK4.1. The residual-stress-related properties of the AlN bulk samples were characterized by LabRAM HR Evolution micro-Raman spectroscopic setup. The room temperature (RT) micro-Raman spectra of bulk AlN samples were recorded under 325 nm and 532 nm excitations, while the temperature-dependent micro-Raman spectra were recorded under 532 nm excitation. In addition, the Linkam liquid nitrogen flowing cryostat (THMS600) was used to vary samples’ temperature from 80 K to ~870 K during the temperature-dependent Raman measurements. All Raman spectra detection was taken under the backscattering configuration with the scattering light parallel to the surface normal direction of AlN bulk, where the polarization of the incident and scattering light were not fitted. The excitation power on the samples was kept very low (<5 mW) to avoid laser heating. The spectral resolution of the micro-Raman setup was 0.2 cm^−1^. All Raman measurements without a polarizer inserted in the optical path were performed on two AlN crystal faces: the c-bulk AlN crystal face with an (0002) orientation along the c-axis (0001) and the m-bulk AlN sample face with an orientation perpendicular to the c-axis. All Raman spectra were collected using the $x\left(-,-\right)\bar{x}$ back-scattering configuration for the m-bulk sample and the $z\left(-,-\right)\bar{z}$ back-scattering configuration for the c-bulk sample.

## 3. Results and Discussion

### 3.1. High-Resolution X-ray Diffraction Analysis

**Figure 1:**
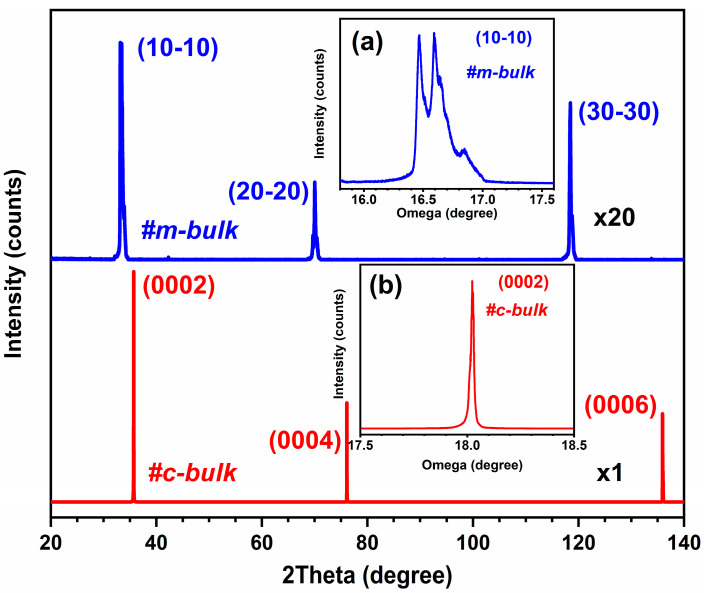
XRD profiles of two bulk AlN crystals, multiplied 20 and 1 times for m-bulk and c-bulk samples, respectively. Inset (**a**) is a rocking curve of (10−10) planes with Omega Rel scan mode of the m-bulk sample. Inset (**b**) is a rocking curve of (0002) planes with Omega Rel scan mode of the c-bulk sample.

High-resolution X-ray diffraction (HR-XRD) is frequently used to characterize the structural and crystalline qualities of crystals. Figure 1 shows the XRD profiles of two bulk AlN crystals from 20° to 140° in the 2θ/ω scanning mode. For the c-bulk sample, the very strong and sharp diffraction peaks of AlN (0002), (0004), and (0006) are observed, indicating that it is a polar c-face AlN crystal, while the XRD profile of the m-bulk sample mainly displays a nonpolar m-face AlN crystal with diffraction peaks of (10−10), (20−20), and (30−30). They belong to the hexagonal wurtzite crystal structure, with a cell volume of 41.724 Å^3^ and lattice constants of a = 3.111 Å, b = 3.111 Å, and c = 4.978 Å [34]. These peaks for m-bulk AlN look a little broader, with shoulders on the left and right side of the main peaks [16]. The broadening of the diffraction peaks may be attributed to the crystalline defects and tilting of the main crystallographic planes that formed the low-angle grain boundaries (LAGBs) during the AlN crystal growth [37]. The X-ray diffraction rocking curves for the asymmetric (10−10) reflection of the m-bulk sample and the symmetric (0002) reflection of the c-bulk sample are shown in insets (a) and (b) of Figure 1, respectively. The inset of Figure 1a shows the slight splitting of the (10−10) peak for the m-bulk sample, indicating that the m-bulk sample has low-angle grain boundaries, its crystal mosaicity is high, and its crystal quality is poor. The splitting of the peak in the rocking curve would be attributed to the inclination of the crystallographic plane produced by the multiple-size expansion process, resulting in the shift of the Bragg diffraction angle. This case is a little similar to that in the epitaxial lateral overgrowth of GaN [38]. The (0002) peak for c-bulk is sharp in Figure 1b, indicating that the crystal quality is good. Moreover, the FWHM values of the asymmetric (10−12) rocking curve for the m-bulk and c-bulk samples are 127.1 and 124.5 arcsec, respectively, indicating that the c-face AlN crystal has a lower dislocation density.

### 3.2. X-ray Photoelectron Spectroscopy Analysis

**Figure 2:**
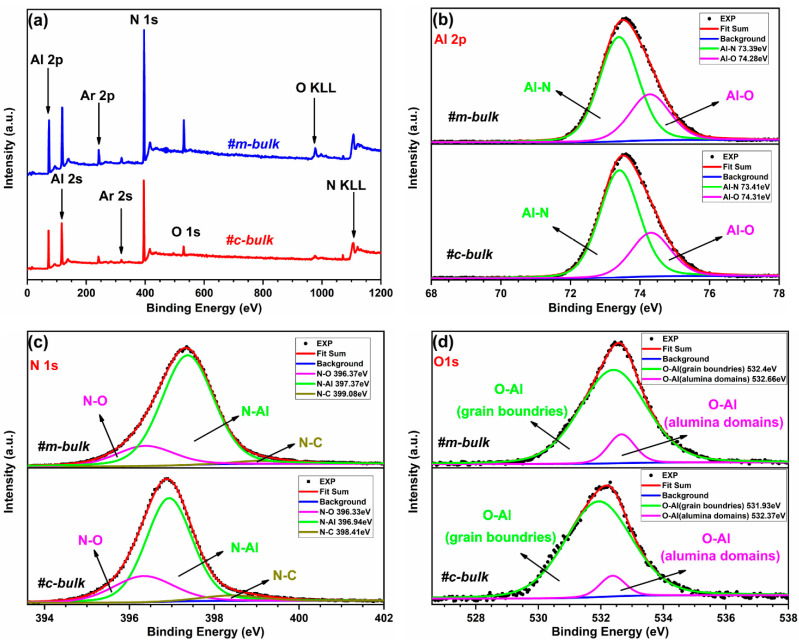
XPS survey spectra (**a**) and fine-scan spectra of (**b**) Al 2p, (**c**) N 1s, and (**d**) O 1s for m-bulk and c-bulk AlN crystals.

The X-ray photoelectron spectroscopy (XPS) is regarded as a powerful and sensitive analytical technique for its high sensitivity to element chemical states. It has been extensively used to probe the surface morphology and composition of AlN and related materials [26,39,40,41]. Figure 2a shows the XPS survey scans of the two bulk AlN crystals, and the characteristic peaks are observed, corresponding to Al (2p, 2s), Ar (2p, 2s), O 1s, N 1s, N KLL (Auger peaks), and O KLL (Auger peaks) in our AlN samples. XPS spectra reveal that the surfaces in the two bulk AlN crystals are composed of the following elements: aluminum, nitrogen, oxygen, and argon. For the m-bulk AlN crystal, the ratio of the atomic concentrations of four elements (Al, N, O, and C) is 47.17:39.45:11.95:1.44 at. %, respectively. For the c-bulk AlN crystal, the atomic ratio for the same four elements is 47.09:41.56:8.67:2.68 at. %, respectively. Carbon mainly comes from the crucible and residual carbon (low content) in the AlN raw materials. Oxygen may be introduced by the sintering process in the chamber, air, or impurities adsorbed on the sample surface. Argon may be introduced during the preparation process. Carbon and oxygen contamination are known to negatively influence crystal properties and quality. Accordingly, the carbon and oxygen content in the m-bulk sample is more than that in the c-bulk sample. Moreover, the stoichiometric portion (Al:N) of the m-plane AlN crystal seems to be better than that of the c-plane AlN crystal, and the over-layers of the m-plane AlN may contain more oxygen impurities and defects that still need to be proven by an XPS depth profiling measurement of the two samples.

The Shirley iterative method was chosen to treat the peak background. A Gaussian–Lorentzian (80%) mixture function was used for the simulation. The function performed results in a fitting analysis of the selected spectra. The fine scan spectra on the Al 2p, N 1s, and O 1s peaks recorded from two bulk AlN crystals are displayed in Figure 2b–d. The relative atomic ratio and bonding phase in the surface region are calculated according to the analyses of the asymmetric shape of the Al 2p, N 1s, and O 1s spectra. All the Al 2p spectra were fitted as two subpeaks of the Al–N bond and the Al–O bond. The binding energies of Al–O are 74.28 eV and 74.31 eV, and the binding energies of Al–N are 73.39 eV and 73.41 eV, for the m-bulk and c-bulk samples, respectively. The nitrogen peak consisted of three subpeaks assigned to three distinct chemical states. The binding energies of N-C are 399.08 eV and 398.41 eV, the binding energies of N-Al are 397.37 eV and 396.94 eV, and the binding energies of N-O are 396.37 eV and 396.33 eV, for the m-bulk and c-bulk samples, respectively. The narrow-spectrum O 1s was deconvoluted into two subpeaks of the O–Al (alumina domains) bond and the O–Al (grain boundaries) bond. The binding energies of O–Al (alumina domains) are 532.66 eV and 532.37 eV, and the binding energies of O–Al (grain boundaries) are 532.4 eV and 531.93 eV, for the m-bulk and c-bulk samples, respectively. These values are in good agreement with the reported results [39,40]. The related chemical state of each subpeak is summarized in Table 1.

The surface oxide thickness *d_xps_*(*nm*) was estimated from the ratio of the area of alumina oxide to the area of alumina nitride (*I_o_*/*I_m_*), as reported in [27,30], so

(1) $$d_{xps}(nm)=\lambda_{o}\sin(\theta )ln\left(\frac{N_{m}I_{o}\lambda_{m}}{N_{o}I_{m}\lambda_{o}}+1\right)$$

where the ratio of the volume densities of the aluminum atoms in metal to oxide is *N_m_/N_o_* = 1.6 (calculated in conformity with the densities of Al = 2.7 g cm^−3^ and Al_2_O_3_ = 3.1 g cm^−3^), *λ_o_* (2.92 nm) and *λ_m_* (2.39 nm) are alumina and the attenuation length of aluminum nitride, respectively, and θ is the take-off angle (45°) [41]. The results of the surface oxide thickness are listed in Table 1.

Table 1 presents a detailed comparison of the XPS fine-scan analyses of the m-bulk and c-bulk samples. This shows that the Al/N ratio of the m-face AlN crystal is bigger than that of the c-face AlN crystal. This can be explained by the correlation between the thicknesses of the oxide layers and Al/N ratios. On account of AlN exhibiting a high oxygen affinity, oxygen contamination is easily formed on the surface [42]. When oxygen atoms entered the AlN lattice, oxygen replaced the nitrogen atoms in the lattice to form aluminum vacancies, and AlN crystals with thicker oxidized surface overlayers have a larger ratio of Al/N. We also can find that the surface oxide thickness for the m-bulk sample is larger than that of the c-bulk sample, as listed in Table 1. Moreover, the disparity in the surface oxide thickness for bulk AlN crystals could be attributed to the difference in oxygen contamination [29], because an oxygen atom tends to enter the lattice of the bulk AlN crystal, while the aluminum atom in the polar crystal prevents the oxygen impurity from entering the lattice to substitute for the nitrogen atom. Contrarily, non-polar AlN exhibited no blocking effect by aluminum atoms on impurities. Consequently, the m-plane AlN crystal could be more easily adversely influenced by oxygen contamination compared to the c-plane AlN crystal.

### 3.3. Raman Spectroscopy Analysis

Raman spectroscopy is a powerful tool for the study of the lattice dynamics of crystals, by giving information about their phonon vibration modes. It is also one of the most efficient, sensitive, and direct techniques due to its non-contact, non-damaging, high-resolution measurement, which can be used for the analysis of semiconductor materials and structures [43,44,45,46,47].

**Figure 3:**
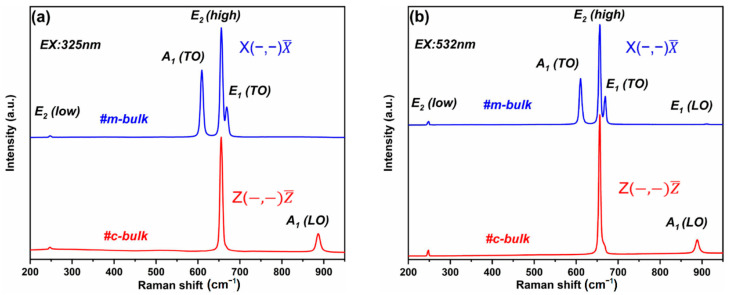
Room temperature Raman spectra excited by (**a**) 325 nm and (**b**) 532 nm laser for two bulk AlN crystals without a polarizer inserted in the optical path.

**Figure A1:**
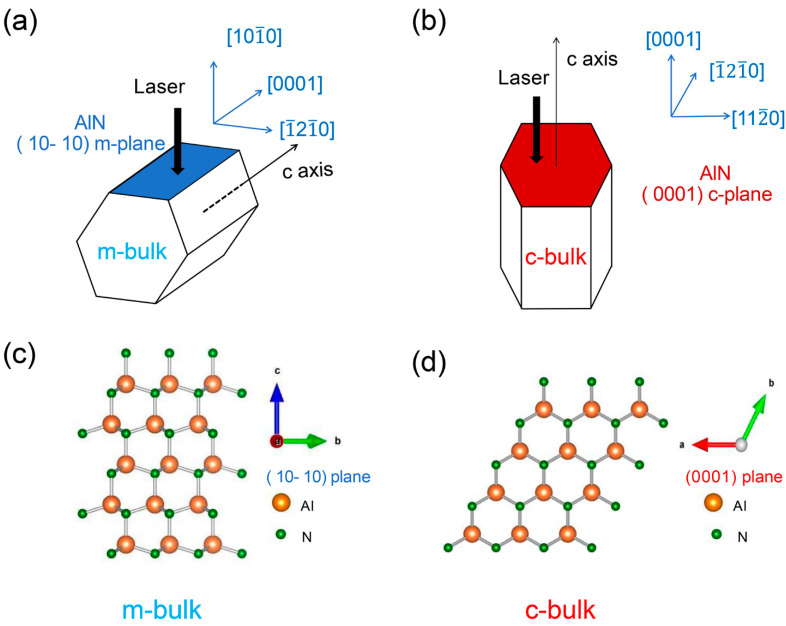
Schematic diagrams of Raman experiments of the m-bulk sample (**a**) and the c-bulk sample (**b**). (**c**) Top view of the crystal structure of the m-bulk AlN crystal in the (10-10) plane perpendicular to the c-axis and (**d**) top view of the crystal structure of the c-bulk AlN crystal in the (0001) plane parallel to the c-axis.

Figure 3 shows the room-temperature Raman spectra of two bulk AlN crystals excited by 325 nm and 532 nm lasers without a polarizer inserted in the optical path. The observed phonon modes in the Raman spectra of the m-bulk and c- bulk AlN wafers obey the C^4^_6V_ point group symmetry rules for the corresponding scattering configuration [44]. According to group theory, eight optical phonon modes are expected at the Γ point: 2A_1_ + 2B + 2E_1_ + 2E_2_. The A_1_ (polarized in the z direction) and E_1_ (polarized in the (x,y) plane) modes are both Raman-active and infrared-active, the E_2_ modes are Raman-active, and the B modes are silent [48]. The A_1_ and E_1_ modes can be split into propagation-parallel longitudinal (LO) and propagation-perpendicular transverse (TO) components, respectively. As shown in Figure A1a,b in Appendix A, the direction of laser incidence is perpendicular to the (10-10) surface for the m-bulk sample, while the direction of laser incidence is perpendicular to the (0001) surface for the c-bulk sample. For the m-bulk sample, the spectra were recorded in the $x\left(-,-\right)\bar{x}$ geometry, which corresponds to the $z\left(-,-\right)\bar{z}$ geometry for the c-bulk sample. In Raman measurement, the backscattering configurations of $x\left(-,-\right)\bar{x}$ and $z\left(-,-\right)\bar{z}$ are to denote the propagation direction parallel to the normal (10-10) AlN surface and (0002) AlN surface with a non-polarized light incidence and non-polarized outgoing light direction, respectively. Considering the direction of phonon propagation, for backscattering from polar AlN, the A_l_ (LO) mode is not allowed in the $x\left(z,y\right)\bar{x}$ and $x\left(y,y\right)\bar{x}$ scattering configurations [49]. For c-plane AlN under backscattering with no polarization detection, it is known that the E_2_ (low), E_2_ (high), and A_1_ (LO) modes are allowed, while the A_1_ (TO) and E_1_ (TO) phonon modes are forbidden to be the Raman selection rules for the $z\left(-,-\right)\bar{z}$ geometry [34]. The Raman spectra revealed that four/five out of the six active Raman modes of the m-bulk sample are observed at 325 nm/532 nm excitation. The peak positions are the E_2_ (low) mode located at about 247.7 cm^−1^/247.6 cm^−1^, the A_1_ (TO) mode located at about 609.2 cm^−1^/610.4 cm^−1^, the E_2_ (high) mode located at about 655.8 cm^−1^/656.1 cm^−1^, the E_1_ (TO) mode located at about 668.9 cm^−1^/669.1 cm^−1^, and the E_1_ (LO) mode located at about 911.2 cm^−1^, which is a normal m-plane AlN Raman spectrum and is in agreement with the formerly reported spectrum [23,44]. For the c-bulk sample, the allowed E_2_ (low), E_2_ (high), and A_1_ (LO) Raman modes are visible near 247.4 cm^−1^/247.3 cm^−1^, 655.5 cm^−1^/656.1 cm^−1^, and 886.7 cm^−1^/888.7 cm^−1^, respectively, all of which are consistent with previously reported peaks [34,50].

The E_2_ (high) peak is extremely sensitive to residual stress, and its frequency shift can be used to estimate the residual stress in AlN crystals. The biaxial stress (*σ*) can be calculated by the following equation [29,30]:

(2) σ=Δω/K

where the difference between the E_2_ (high) phonon frequency for the AlN crystals of the unstressed position (*ω*_0_) and stressed position (*ω*) is Δ*ω* = *ω*_0_ − *ω*, with *K* (2.4 ± 0.2 cm^−1^ GPa^−1^) [47] being the strain coefficient, and *ω*_0_ (657.4 ± 0.2 cm^−1^) being the unstressed E_2_ (high) phonon frequency of the AlN crystal [35,47]. With increasing compressive biaxial stress, the E_2_ (high) phonon frequencies of the AlN crystals increase in comparison with the stress-free position, while increasing tensile stress corresponds to the E_2_ (high) phonon frequencies decrease concerning the unstressed position [51,52]. The FWHM and Raman frequency positions of the E_2_ (high) phonon mode fitted by the Voigt function and the calculated results of stress are listed in Table 2 for the m-bulk and c-bulk crystals. Obviously, the FWHM of the m-bulk sample is larger than that of the c-bulk sample. With excitation laser sources of both 325 nm and 532 nm wavelengths, it is observed that the FWHM of the m-bulk AlN sample is larger than that of the c-bulk AlN. The linewidth of the E_2_ (high) peak of AlN crystals increases with an increase in defects and dislocations. Therefore, the crystal quality of the m-face AlN crystal is not as good as that of the c-face AlN crystal, implying that the defect density of the m-face AlN crystal is possibly higher than that of the c-face AlN crystal. This result is consistent with the XRD analysis. At the same time, both crystals exhibit tensile residual stresses at room temperature, though the c-bulk sample shows more residual stress than the m-bulk sample. Given that the laser penetration depth is related to its wavelength, the 325 nm laser has a smaller penetration depth than the 532 nm laser. Hence, the 325 nm laser can only probe the near-surface region of the AlN crystals, while the 532 nm laser may probe the deeper region of the samples. The biaxial stress of the two samples excited by the 532 nm laser is less than that excited by the 325 nm laser, indicating that the surface of the crystals has more residual stress than the inside of the crystals. Interestingly, the residual stress of the c-face AlN crystal is much greater than that of the m-face AlN crystal, and the surface stress in both samples is larger than the stress inside them. Maybe this effect is related to the crystal structure and growth process, but the exact cause is not yet clear and needs to be further investigated.

### 3.4. Temperature-Dependent Raman Analysis

**Figure 4:**
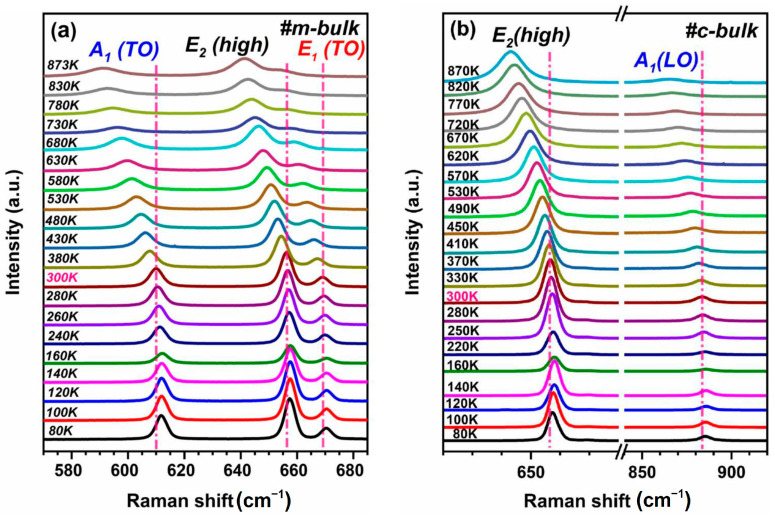
Raman spectra (**a**,**b**) for m-bulk and c-bulk samples excited by 532 nm laser were taken at different temperatures from 80 to nearly 870 K.

Temperature-dependent Raman scattering can be employed to probe the temperature effect of bulk AlN crystal [29,30,31]. Figure 4 shows the temperature dependence of the Raman spectra for m-bulk and c-bulk AlN crystals excited by the 532 nm laser.

For m-bulk and c-bulk crystals, the frequencies of the Raman phonon modes tend to shift toward lower frequencies, while the FWHM values of the peaks gradually increase with increasing temperature from 300 K to ~870 K; however, the Raman peaks of the two bulk AlN crystals almost do not have any noticeable change in the lower temperature range (80 K–300 K). For example, the peak positions of the E_2_ (high) of the m-bulk sample are 657.4 cm^−1^, 656.2 cm^−1^, and 641.3 cm^−1^ at 80 K, 300 K, and 873 K, respectively. Similarly, the frequencies of the E_2_ (high) of the c-bulk sample are 657.5 cm^−1^, 656.6 cm^−1^, and 643.1 cm^−1^ at 80 K, 300 K, and 870 K, respectively. Firstly, the temperature dependence of the Raman frequency shift is related to the change in the lattice vibration frequency, which is caused by either the thermal expansion or contraction of the lattice or the anharmonic effect of the lattice vibration resulting from the process of a higher energy optical phonon decaying into lower energy phonons. Secondly, the temperature dependence of the linewidth could mainly be explained by the phonon–phonon scattering. Finally, the Raman shift of the E_2_ mode can reflect the variation of the residual stress in the crystals.

The T-variable Raman spectra of AlN may be fitted with the Voigt profile by Equation (3) [53]:

(3) $$\omega (T)=\omega_{0}-A/\left\{\exp\left[B\left(hc\omega_{0}/k_{B}T\right)\right]-1\right\}$$

**Figure 5:**
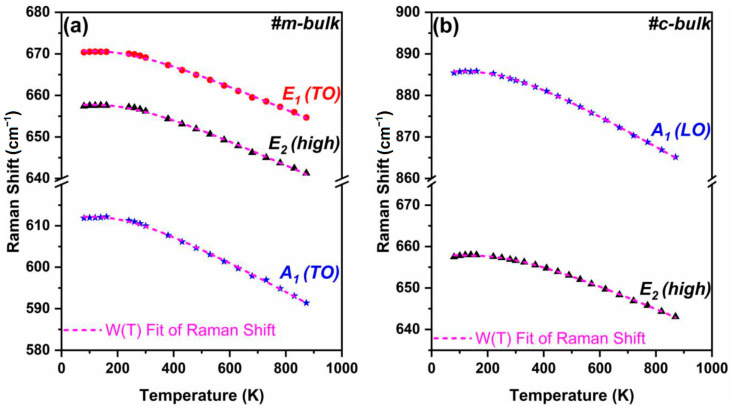
Raman shift (**a**,**b**) for m-bulk and c-bulk AlN crystals as a function of temperature.

At higher temperatures, Equation (3) can be simplified to [53]

(4) $$\Delta \omega =-(Ak_{B}/Bhc\omega_{0})\Delta T$$

where ∆*ω* is the shift of the phonon frequency, ∆*T* is the change in temperature, and *Ak_B_*/*Bhcω*_0_ serves as a frequency–temperature coefficient. The fitted results are shown in Figure 5, and they are well-matched with the temperature dependencies of all the AlN Raman phonon frequencies. The fitted parameters of *ω*_0_, *A*, *B*, and *Ak_B_*/*Bhcω*_0_ are tabulated in Table 3 for all the Raman-active modes of bulk AlN. The Raman frequency shifts of the E_2_ (high) phonon of the m-bulk and c-bulk AlN crystals are 16.1 cm^−1^ and 14.4 cm^−1^, with the increased temperature in the range of 80-nearly 870 K, respectively. Moreover, concerning the ordering of the *Ak_B_*/*Bhcω*_0_ values of the E_2_ (high) phonon mode, the m-bulk sample has larger values than the c-bulk sample. Therefore, the Raman frequency shift of the E_2_ (high) phonon mode of the m-AlN crystal is affected by temperature more significantly than that of the c-AlN crystal.

**Figure 6:**
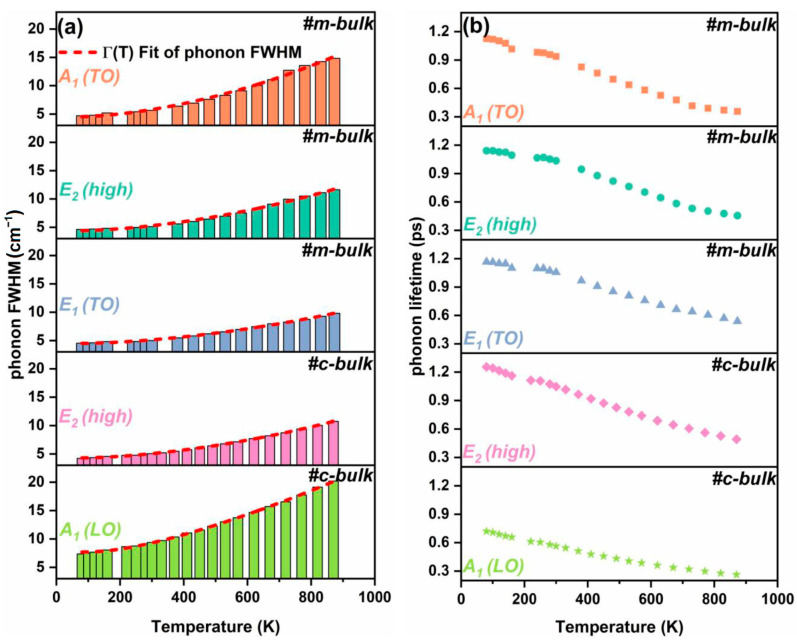
Phonon FWHM (**a**) and phonon lifetime (**b**) of m-bulk and c-bulk samples as a function of temperature.

Phonon broadening is mainly caused by the anharmonic decay of zone-center optical phonons into zone-edge acoustic phonons, the inhomogeneous scattering of phonons by defects, and the confinement of phonons (which is not expected). Assuming that phonon damping mainly arises from the anharmonic effects and symmetrically decays into two and three phonons, the phonon linewidth with a variable temperature can be fitted by the following relationship [48]:

(5) $$\Gamma (T)=\Gamma (0)+C[1+2n(\omega_{0}/2,T)]+D[1+3n(\omega_{0}/3,T)+3n^{2}(\omega_{0}/3,T)]$$

where *n*(*ω*,*T*) = [exp(*hcω*/*k_B_T*) − 1]^−1^ is the Bose function at energy *hcω*, *C* and *D* are constants, Γ(0) +C+D is the FWHM value at 0 K, *ω*_0_ corresponds to the Raman frequency at 0 K, and Γ(0) denotes a damping contribution due to defect or impurity scattering. The short dashed lines in Figure 6a are the best fits using Equation (5), and the fitting parameters are summarized in Table 3. It is clearly found that the FWHM values of the Raman active modes increase with the increased temperature. The E_1_ (TO) phonon mode has the narrowest band, followed by the E_2_ (high) phonon mode and A_1_ (TO) phonon mode, while the A_1_ (LO) phonon mode is considerably broader. From the ratio of C/D, the relative contributions of the second-order and third-order processes to the total phonon decay can be estimated. These ratios of the TO-phonon in two bulk AlN crystals are more than 1, indicating that the decay into two phonons is the dominant process. Meanwhile, the linewidth and ratio of the *C*/*D* of the A_1_ (LO) phonon are much larger than those of the E_2_ (high) phonon, possibly indicating that there are more channels for the decay of the LO-phonon than for the decay of the TO-phonon for the c-bulk sample. In addition, at the same temperature, the E_2_ (high) phonon FWHM of the m-bulk sample is always larger than that of the c-bulk sample. The increase in the E_2_ (high) phonon FWHM of the m-plane AlN sample (7.0016 cm^−1^) is obviously different from that of the c-plane AlN sample (6.5536 cm^−1^), which is possibly caused by the different factors in the two samples including the material boundaries and point defects that originated from the thermal mismatch and alignment disorder.

The phonon lifetime, *τ*, can be estimated from the corresponding damping constant by using the energy–time uncertainty relation [54],

(6) $$\frac{\Gamma }{\hbar }=\frac{1}{\tau }$$

Here, Γ is the line width of phonons in units of cm^−1^, and ℏ (=5.3 × 10^−12^ cm^−1^·s) is the Planck constant. We calculate the phonon lifetime and acquire the relationship between the Raman phonon lifetime of the samples and temperature, as illustrated in Figure 6b. The main contributions to the total phonon lifetime, *τ_total_*, come from the anharmonic decay, *τ_a_*, and the inhomogeneous impurity phonon scattering, *τ_d_*, according to [55]:

(7) $$\tau_{total}^{-1}=\tau_{a}^{-1}+\tau_{d}^{-1}$$

The basic mechanism affecting the lifetime of phonons is the phonon anharmonic decay, such that energy and momentum are conserved in the process. According to Equations (5)–(7), the fitting results using Equation (6) may also be used to obtain information about the impurity-related phonon lifetime, *τ_d_*, as shown in Table 3. The first term in Equation (5) could be attributed to the influence of the impurity and defect phonon scattering processes, *τ_d_*^−1^.

Figure 6a shows the trend of the FWHM values of the Raman modes, and they increase with elevated temperatures for the two samples. The FWHM values of the A_1_ (LO) mode vary more remarkably than those of the E_2_ (high) and E_1_ (TO) modes for the c-bulk sample, while more changes occur to the A_1_ (TO) mode than the E_2_ (high) and E_1_ (TO) modes for the m-bulk sample. The phonon lifetime gradually decreases with the increased temperature, as shown in Figure 6b. As the temperature increases, a harmonic interaction between the phonons increases, and the likelihood of a scattering event increases accordingly. In other words, the phonon decay and phonon damping increase, while the phonon scattering time and the phonon lifetime decrease.

On the contrary, the phonon lifetimes show a different tendency from their FWHM values. The lifetime of the A_1_ (LO) phonon mode of the polar c-plane AlN is the shortest among the phonon modes studied here. Compared with the TO-phonon modes, the A_1_ (LO) phonon may be more easily affected by other factors, such as temperature-dependent carrier density and plasma damping. Thus, the Raman shift of the A_1_ (LO) phonon is not applied to determine the temperature of the sample. On the one hand, the damping contribution of the TO-phonon modes caused by defect or impurity scattering is almost similar in the m-plane AlN. On the other hand, for the c-plane AlN, the damping contribution of the impurity or defect phonon scattering of the A_1_ (LO) phonon modes is more significant than that of the E_2_ (high) phonon. Moreover, the lifetime of the A_1_ (LO) phonon mode is shorter than that of the E_2_ (high) phonon mode in the c-plane AlN. Therefore, the influence of anharmonic decay for the E_2_ (high) phonon lifetime is stronger than that for the A_1_ (LO) phonon mode for the c-plane AlN.

**Figure 7:**
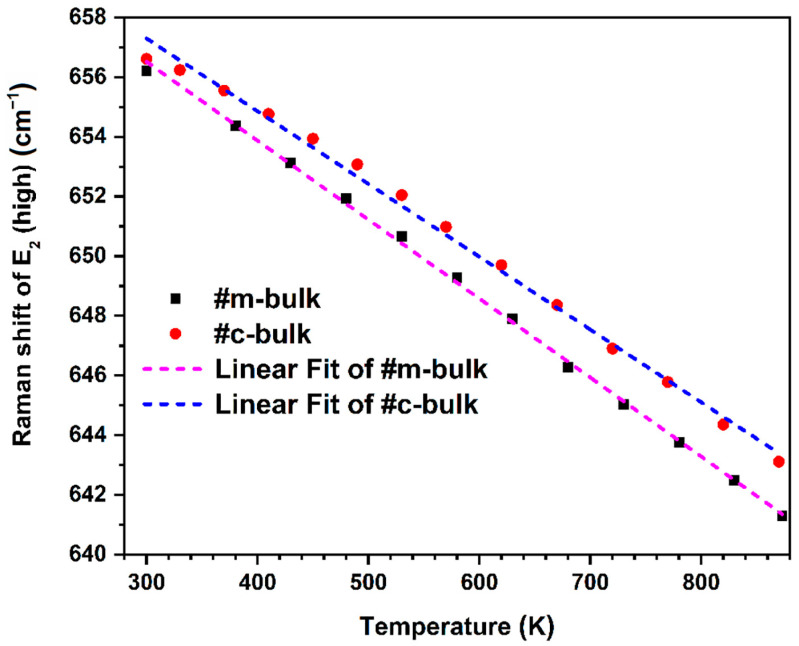
E_2_ (high) phonon shifts of samples as a function of temperature at high temperature.

In the higher temperature range from 300 K to nearly 870 K, as illustrated in Figure 7, the temperature dependency of the Raman shifts of the E_2_ Raman peaks are found to follow linear approximation and can be well-expressed by

(8) $$\omega =\omega_{0}+AT$$

where *ω*_0_ is the harmonic frequency, and *A* is the calibration constant. The fitting results are shown in Figure 7 and Table 4. The slope for the m-face AlN crystal is a little larger than that for the c-face AlN crystal in a higher temperature range, which is in agreement with the result that the m-face AlN is more easily affected by temperature than the c-face AlN.

The temperature dependence of the phonon frequency follows anharmonic terms in the vibrational Hamiltonian of the crystal lattice. Heating of the lattice results in lattice dilation or volume expansion, and then the restoring force of the lattice vibration decreases. Hence, the phonon vibrational frequency was shifted. Besides the effect of thermal expansion, as the temperature gradually increases, the non-intermittent coupling between the phonons increases. The temperature-dependent phonon frequency shift Δ*ω*(*T*) is written as [56]

(9) $$\Delta \omega (T)=\omega (T)-\omega_{0}=\Delta \omega_{e}(T)+\Delta \omega_{d}(T)$$

where *ω*(*T*) is the Raman shift at temperature *T*, *ω*_0_ is the harmonic frequency of the phonon mode, ∆*ω_e_*(*T*) is the thermal expansion contribution to the frequency shift, and ∆*ω_d_*(*T*) denotes the frequency shift due to the anharmonic interaction term. The term ∆*ω_e_*(*T*) is given by [57]

(10) $$\Delta \omega_{e}(T)=-\omega_{0}\gamma \int_{0}^{T}\left[\alpha_{c}(\tilde{T})+2\alpha_{a}(\tilde{T})\right]d(\tilde{T})$$

where *α_c_* and *α_a_* are the temperature-dependent coefficients of the linear thermal expansion that is parallel and perpendicular to the hexagonal c axis, respectively, and *γ* is the Gruneisen parameter. The term ∆*ω_d_*(*T*) is written as [58]

(11) $$\Delta \omega_{d}(T)=A\left[1+2n(T,\omega_{0}/2)\right]+B\left[1+3n(T,\omega_{0}/3)+3n^{2}(T,\omega_{0}/3)\right]$$

(12) $$n(T,\omega )={\left[\exp(\hbar \omega /k_{B}T)-1\right]}^{-1}$$

where Equation (12) is the Bose–Einstein distribution function, and A and B are the anharmonic decay constants. Adopting *ω_0_*, as calculated in Table 4, and the published results of *α_c_* [59], *α_a_* [59], and *γ* [60] for the E_2_ (high) phonon mode, ∆*ω_e_*(*T*) can be calculated without any fitting parameters. Equation (10) can be written as Δ*ω*(*T*) = ∆*ω_e_*(*T*) = −0.02612*T* for the m-plane AlN and c-plane AlN, which is close to the linearly fitted results in the higher temperature range. Therefore, in the higher temperature region, the phonon frequency shift is strongly influenced by lattice expansion with temperature change, while the effect of the decay of the optical phonons into the lower energy side can be neglected. Moreover, it is known that the E_2_ (high) phonon, with its high Raman scattering cross-section, is unaffected by changes in the free carrier concentration. Therefore, the E_2_ (high) phonon may be applied to characterize related aluminum nitride materials in the case of there being no information about the aluminum composition.

**Figure 8:**
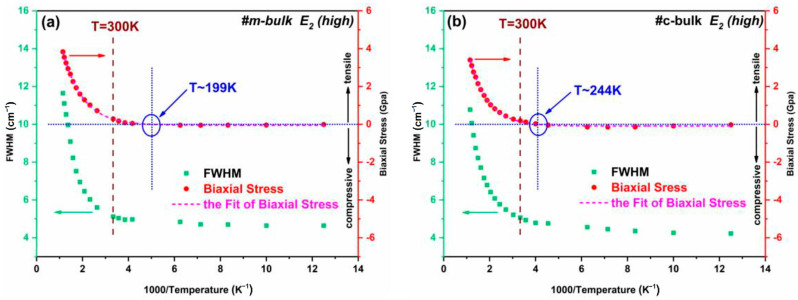
Relationships (**a**,**b**) between the biaxial stress and FWHM of E_2_ (high) mode versus 1000/T for the m-bulk and c-bulk samples.

The E_2_ (high) mode frequency shift of AlN crystals is often used to calculate the residual stress by using Equation (2). In order to analyze the influence of temperature on biaxial stress, we plot the biaxial stress and FWHM of the E_2_ (high) phonon mode versus 1000/T, and the results are shown in Figure 8. It is evident that the biaxial stress of the m-bulk and c-bulk samples has a similar trend with 1000/T increasing, indicating that there exists an identical mechanism in the two AlN crystals. The tendency between the biaxial stress and temperature could be regarded as the first-order exponential growth mode, *σ*(*T*) = *σ*_0_ + *A*exp(1/(*tT*)), where *k* = 1/*t* is the temperature-dependent stress growth rate. The fitted parameters of *σ*_0_, *A*, and 1/*t* are tabulated in Table 5.

At the lowest temperature, the FWHM values of the E_2_ (high) mode of the two AlN crystals are the smallest, corresponding to their biaxial stress being compressive stress, while at the highest temperature, they show the largest half-width and present tensile stress. In contrast to the lower temperature region, the FWHM values of the E_2_ (high) mode and biaxial stress of the two AlN crystals in the higher temperature region exhibit noticeable changes. Moreover, from 80 K to ~870 K, the stress of the two AlN crystals first increases, then decreases, and finally increases with the increase in temperature. However, at a certain temperature, the biaxial stress of the AlN crystal changes from compressive stress to tensile stress, that is, the value of *σ* changes from negative to positive. The stress-transformed temperature is estimated to be 199 K and 244 K for the m-plane AlN and c-plane AlN samples studied here based on the fitting curves (without excluding the interference of instruments), respectively. This conversion temperature is probably related to the crystalline perfection of the samples. In addition, the value of *dσ*/*dT* for the two bulk AlN crystals under tensile stress is investigated. The larger the value of *dσ*/*dT* is, the worse the quality of the sample is, and the phonon frequency shift can be affected more easily by increasing temperature. When samples are under tensile stress, the value of *dσ*/*dT* for the m-bulk AlN is always larger than that for the c-bulk AlN with the increase in temperature, indicating that the m-face AlN sample has a larger stress growth velocity change with the increased temperature than the c-face AlN sample.

## 4. Conclusions

The surface, structural, and optical properties of two aluminum nitride bulks grown by metal system physical vapor transport (PVT) and the effect of temperature on their stress and optical properties were studied. The XRD results show that the m-bulk sample is mainly an m-oriented bulk crystal, but the c-bulk sample is a c-oriented bulk crystal. The two bulk AlN crystals contain aluminum oxide besides Al-N at the surface, as probed by XPS. The surface oxide thickness of the c-plane AlN sample is thinner than that of the m-plane AlN sample. The frequency shift and FWHM of the Raman-active modes for the two bulk AlN crystals roughly increase with the increased temperature, while the phonon lifetime gradually decreases. The Raman frequency shift of the E_2_ (high) phonon mode of the m-face AlN crystal is more easily affected by the temperature compared with that of the c-face AlN crystal. For both AlN crystals, the decay of all the Raman-active modes into two phonons is the prevailing process. We also can obtain information about the impurity-related phonon lifetime from the Raman FWHM. The lifetime of the TO-phonon mode is less than that of the LO-phonon mode affected by crystal imperfections, which is attributed to the stronger anharmonic effect on the former. In the higher temperature region, the relation between the Raman shift and temperature is approximately linear, and the phonon frequency shift is strongly influenced by lattice expansion with increasing temperature. Meanwhile, the room-temperature Raman spectra displayed that the FWHM of the m-bulk sample is larger than that of the c-bulk sample, whereas the residual stress in the m-bulk sample is shorter than that in the c-bulk sample. In addition, the samples both have an inhomogeneous strain, and the stress of the surface layer is larger than that inside the sample. We also discussed the influence of temperature on stress. We found that the biaxial stress of AlN crystal changes from compressive stress to tensile stress at a certain temperature.

## Figures and Tables

**Table 1:** XPS analysis result for two bulk AlN crystals.

Sample #			m-bulk	c-bulk
Al 2p	#1 Al-O	B.E. (eV)	74.28	74.31
		Area	8660.721	8045.585
		FWHM	1.367	1.347
	#2 Al-N	B.E. (eV)	73.39	73.41
		Area	19,014.39	19,631.26
		FWHM	1.325	1.335
N 1s	#1 N-C	B.E. (eV)	399.08	398.41
		Area	2568.217	3256.731
		FWHM	1.349	1.639
	#2 N-Al	B.E. (eV)	397.37	396.94
		Area	95,942.13	48,926.52
		FWHM	1.445	1.289
	#3 N-O	B.E. (eV)	396.37	396.33
		Area	19,656.24	16,183.78
		FWHM	1.609	1.680
O 1s	#1 O-Al (alumina domains)	B.E. (eV)	532.66	532.37
		Area	6469.499	1694.481
		FWHM	1.008	0.96
	#2 O-Al (grain boundaries)	B.E. (eV)	532.4	531.93
		Area	51,955.99	20,589.39
		FWHM	2.508	2.52
AlN_Al_/AlN_N_			1.327	1.124
d_xps_(nm)			0.966	0.887

**Table 2:** The full width at half maximum (FWHM) and biaxial stress of the E_2_ (high) peak of two bulk AlN crystals at RT.

Sample #		m-bulk	c-bulk
325 Raman	FWHM (cm^−1^) of E_2_ mode	7.4	7.2
	*σ* (GPa)	0.326 ± 0.011	0.462 ± 0.014
532 Raman	FWHM (cm^−1^) of E_2_ mode	5.4	5.2
	*σ* (GPa)	0.307 ± 0.004	0.319 ± 0.025

**Table 3:** Parameters *ω*_0_, *A*, *B*, *Ak_B_*/*Bhcω*_0_, *C*, *D*, Γ(0), *C*/*D*, and *τ_d_* for the phonons of two bulk AlN crystals.

Sample #	m-bulk			c-bulk	
	A_1_ (TO)	E_2_ (High)	E_1_ (TO)	E_2_ (High)	A_1_ (LO)
*ω*_0_ (cm^−1^)	612.2 ± 0.1	657.7 ± 0.1	670.7 ± 0.1	657.9 ± 0.1	885.8 ± 0.1
*A* (cm^−1^)	29.9 ± 1.9	27.0 ± 1.9	24.5 ± 1.7	30.8 ± 1.3	36.0 ± 1.1
*B*	0.8818 ± 0.0375	0.8845 ± 0.0405	0.8357 ± 0.0381	1.0256 ± 0.0263	0.6850 ± 0.0136
*Ak_B_*/*Bhcω*_0_ (×10^−2^ cm^−1^ K^−1^)	3.846	3.223	3.035	3.170	4.120
*C*	0.4786 ± 0.0010	0.3819 ± 0.0009	0.3200 ± 0.1562	0.3370 ± 0.0688	4.4060 ± 0.4414
*D*	0.3506 ± 0.0076	0.2766 ± 0.0064	0.2052 ± 0.0203	0.2465 ± 0.0088	0.3489 ± 0.0697
*C*/*D*	1.4 ± 0.0	1.4 ± 0.0	1.6 ± 0.8	1.4 ± 0.3	12.6 ± 2.8
Γ(0) (cm^−1^)	3.6954 ± 0.096	3.7488 ± 0.0704	3.9696 ± 0.1628	3.7032 ± 0.0731	2.9366 ± 0.4051
*τ_d_* (ps)	1.434 ± 0.007	1.414 ± 0.005	1.335 ± 0.010	1.431 ± 0.005	1.805 ± 0.050

**Table 4:** Fitting parameters for the relations between E_2_ (high) phonon shift and temperature of two bulk AlN samples by linear function at high temperature.

Sample #	m-bulk	c-bulk
*ω*_0_ (cm^−1^)	664.5 ± 0.2	664.6 ± 0.3
*A* (cm^−1^ K^−1^)	−0.02646 ± 0.00028	−0.02439 ± 0.00049

**Table 5:** Fitting parameters *σ_0_*, *A*, and 1/*t* for the relationship between E_2_ (high) phonon biaxial stress and temperature of two bulk AlN samples by the first-order exponential growth function.

Sample #	m-bulk	c-bulk
*σ*_0_ (×10^−3^ Gpa)	−48.46 ± 11.40	−88.82 ± 13.20
*A* (Gpa)	14.05 ± 0.25	14.47 ± 0.37
*k* (×10^3^ K)	−1.13 ± 0.01	−1.24 ± 0.02

## Data Availability

The data that support the findings of this study are available from the leading author, S.Z., upon reasonable request.
